# Influences and Implications of Medical Mistrust on Healthcare Behaviors in a Low Health Outcomes County in the State of New Jersey

**DOI:** 10.1007/s10900-025-01483-5

**Published:** 2025-05-27

**Authors:** Dale Johnson, Adeena Javed, Nathaniel J. Byrnes, Anne C. Jones, Kristin N. Bertsch

**Affiliations:** Department of Family Medicine, Rowan-Virtua School of Osteopathic Medicine, Stratford, NJ USA

**Keywords:** Trust, Information seeking behavior, Health behavior, Public health, Preventative medicine

## Abstract

Medical mistrust is an ongoing concern in the United States, with patient confidence in both individual practitioners and institutions decreasing sharply in recent years. Atlantic County, New Jersey has some of the worst health outcomes in the state and is below the national average. This study sought to investigate medical mistrust by recruiting participants to complete a survey and/or health screening at 12 food pantry distribution events throughout Atlantic County (*N* = 124). The mean score for the Medical Mistrust Index was 19.06 (*SD* = 5.6) for participants who reported receiving the annual influenza vaccine and 16.05 (*SD* = 4.7) for participants who did not report receiving the vaccine (*p* = 0.002). Participants who reported an “Excellent” self-perception of overall (*M* = 13.7, *SD* = 4.6) and dental health (*M* = 14.6, *SD* = 5.5) had significantly less trust relative to their counterparts. There was no significant difference in self-perception of mental health and trust. While not statistically significant, a positive correlation was observed between trust and the number of healthcare information sources used: participants reporting use of 1 source had the lowest trust (*M* = 16.82, *SD* = 5.73), while those using 2 (*M* = 17.86, *SD* = 4.48; *p* =.509), 3 (*M* = 17.33, *SD* = 0.87; *p* =.536), or ≥ 4 sources (*M* = 19.38, *SD* = 3.97; *p* =.086) showed progressively higher trust. Our findings highlight the responsibility of improving medical mistrust falls on both providers and patients to take agency of their care.

**Supplementary Information:**

The online version contains supplementary material available at 10.1007/s10900-025-01483-5.

## Introduction

Of counties in southern New Jersey (NJ), Atlantic County has among the highest rates of substance abuse, diabetes, obesity, and asthma based on data from the NJ State Department of Health [1, 2]. While the U.S. Census provides data down to the census block level, community needs assessment surveys are crucial tools for capturing subtle findings at a granular level [3]. Trust in health systems and healthcare clinicians themselves is essential to enabling adequate engagement with available resources [4]. Current research on medical mistrust focuses on non-modifiable demographic factors that are correlated with behavior, such as age, race/ethnicity, and gender [5]. Furthermore, common study populations have limited, adequate exploration of the relationship between low socioeconomic status and medical mistrust [6], despite the recognized negative health consequences of low socioeconomic status as a social determinant of health (SDoH) [7].

According to the COVID States Project [8], a U.S. nationwide survey, medical mistrust is a growing concern with some researchers regarding it as a SDoH that has psychosocial implications, particularly on medically underserved areas [9]. Those reporting “a lot of trust in physicians and hospitals” sharply decreased from 71.5% to 40.1% from April 2020 to January 2024 [10]. Medical mistrust has been shown to have multiple deleterious effects across the continuum of an individual's healthcare, including: the seeking out of services [11], where services are received [12], adherence [13], and perception of care received [14]. Using the Medical Mistrust Index (MMI), among a variety of demographic characteristics, mistrust impacted the greatest number of barriers to healthcare including: taking medical advice, keeping follow-up appointments, and postponing seeking required care [15]. Other demographic factors, such as education, income, insurance status of Medicaid/Medicare, lack of insurance, and age were only correlated with 2 or fewer of the 5 barriers investigated, while race and gender were associated with none [15]. Through mediation analysis, another study found that among race/ethnicity, perceived discrimination, and medical mistrust, only medical mistrust was significantly associated with vaccination behavior, having a negative correlation [16]. Pertaining to the COVID-19 vaccine specifically, research suggests improving medical mistrust by addressing causes of medical mistrust rather than correcting individuals’ beliefs and knowledge may be most efficacious [5]. Accumulating research suggests focusing this framework on government institutions, not individual providers [17]. Conversely, in a review of internet health informationseeking behaviors, increased information seeking by patients had a strong, direct relationship with patient-physician relationship and confidence in treatment plans [18].

The purpose of this research is to build on previous studies in addressing the need for greater understanding of the relationship between medical mistrust and health care behaviors. Specifically, this paper investigates the relationship of medical mistrust and participant demographics, information seeking behaviors, preference of healthcare location to seek services, self-perception of health (overall, dental, and mental), annual flu/COVID vaccination, and annual hypertension/cholesterol screening within a lower socioeconomic status, rural community in southern NJ.

## Method

### Procedure

The present study was conducted as a subset of a larger community needs assessment for residents of Atlantic County, New Jersey which evaluated regional differences in healthcare utilization, perceived needs of healthcare services, SDoH factors, and medical trust. Data collection took place during food distribution events at 6 locations within Atlantic County across 12 events from September 2023 to June 2024. Events were organized and overseen by either the Atlantic County Sheriff’s Department, Community Food Bank of New Jersey, or Mighty Writers, receiving donations of fresh produce and nonperishable items to distribute on a first-come, first-served basis. Advertisements for distribution events appeared on each organization's website and/or social media outlets. Food donations were available and accessible to all who attended the event. Volunteers from the community, local spiritual organizations, medical schools, and service organizations attended to distribute the food in an organized fashion. Locations were selected to capture diverse populations across both rural and urban areas of the county and leverage the benefits of well-established social services [19, 20]. Only participants who completed all items within the MMI scale were included in the present analysis (*N* = 124).

### Measures

#### Health Screening

Trained medical students were provided oversight by a family medicine attending/faculty member and/or a senior resident. Screening metrics included measurements of blood pressure (Welch Allyn, Skaneateles Falls, New York) [21] and blood glucose levels (Trividia Health, Fort Lauderdale, Florida) [22].

#### Survey

A comprehensive online survey was developed to assess various aspects of health and SDoH. The survey consisted of approximately 70 questions and was available in both English and Spanish to accommodate participants’ language preferences. Participants had the choice to use an iPad provided by the researchers or scan QR codes that allowed participants to use their personal devices to complete the survey. Key domains of the survey included:

1. Healthcare Trust: Using the validated, abridged form [15, 23] of the MMI, shown in Table S1 of the online supplement, we assessed attitudes toward healthcare trust using a 5-point Likert scale based on agreement with the statement (1 = “Strongly Agree”, 3 = “Neutral”, and 5 = “Strongly Disagree”). Mistrust was scored based on the sum of the responses to each item in the scale (range = 7 to 35) where higher scores indicate greater trust.
2. Demographics: Gender, age, language, education, income, and insurance status.
3. Information Seeking Behaviors: Source of healthcare information (specific sources and total number reported to be used).
4. Personal Health: Primary location of seeking healthcare services. Self-perception of overall, dental, and mental health.
5. Preventative Measures: Self reported annual flu and COVID vaccination, hypertension screening, and cholesterol screening. Blood pressure and sugars measured through the health screening we provided.

### Participants

Participants were recruited on-site at food distribution events. Trained medical students, fluent in English and Spanish, walked up and down the car lineups to recruit patients to complete the survey and screening. Eligibility criteria included being a resident of Atlantic County and being aged 18 years or older. Demographics of the sample population are found in Table 1 below.

**Table 1:** Demographics of survey participants

Demographic variable	Participant completion		Average value (± *SD*)
	*n*	%	
*Medical Mistrust*	124	100	17.2 (*SD* = 5.2), Median = 18
*Gender*	116		
Male	31	26.7	
Female	85	73.3	
*Age*	89		54.6 (*SD* = 15.3)
18–30	8	9.0	
31–50	21	23.6	
51–70	52	58.4	
> 70	8	9.0	
*Survey Language*	124		
English	105	84.7	
Spanish	19	15.3	
Education	97		
High school or less	53	54.6	
Some college or more	44	45.4	
*Income*	89		Median = $20,000—$29,999
≤ $19,999	38	42.7	
$20,000- $49,999	39	43.8	
$50,000- $79,999	8	9.0	
≥ $80,000	4	4.5	
Health Insurance*	124		
Private	30	24.2	
Medicare/Medicaid, Federal Funded	83	67.0	
Uninsured	6	4.8	
Other/I don’t know	11	8.9	
*Perception of…*			
Overall health	124	100	Excellent (*n* = 16), 12.7% Very Good (*n* = 21), 16.7% Good (*n* = 47), 37.3% Fair (*n* = 32) = 25.4% Poor (*n* = 8), 6.3%
Dental health	124	100	Excellent (*n* = 22), 17.5% Very Good (*n* = 23), 18.3% Good (*n* = 30), 23.8% Fair (*n* = 27) = 21.4% Poor (*n* = 22), 17.5%
Mental health	121	97.6	Excellent (*n* = 34), 27.0% Very Good (*n* = 26), 20.6% Good (*n* = 37), 29.4% Fair (*n* = 15) = 11.9% Poor (*n* = 9), 7.1%

*Participants were able to select all that apply to themselves and those in their household. The vast majority of participants selected only 1 form of coverage, 6 participants selected 2 options (5 of which were private and Medicare/Medicaid option)

### Data Collection and Quality Control

Surveys were administered in person by faculty, physicians, resident physicians, and medical students trained in culturally sensitive practices. Participants were given the option to complete the survey independently or with assistance. Once data collection concluded, the data were screened to exclude responses which did not give consent to be included in the study, answer more than the first 5 questions, live in the county, or provide a ZIP code to verify they were a resident of Atlantic County. Subsequently, only those who completed all medical mistrust questions were included for final analysis.

### Statistical Analysis

Quantitative data analysis was conducted using IBM SPSS Statistics (Version 29) software and aggregate demographic factors were tabulated (Table 1) and characterized by mean, median, and provided distribution where appropriate. Internal consistency of the MMI scale was assessed using Cronbach’s α to determine if any items should be removed. Using all 7 items, there was a Cronbach's α of 0.85, demonstrating good internal consistency. Consistency was highest with all 7 items and therefore all 7 were used in subsequent analyses. Due to the non-normal distribution of the MMI data, additional analyses applied nonparametric tests, Mann–Whitney U or Kruskal–Wallis.

### Compensation & Ethical Considerations

Hygiene kits were distributed to any participant who completed either portion of the study. Participants were also offered a $10 e-gift card or a backpack with hygiene products as compensation for completion of the survey and/or the health screening for a total potential compensation of $20, or the alternative backpack compensation. The development of the Spanish survey offering was in congruence with Institutional Review Board (IRB) standards which entailed translation performed by one party and then verified by another, independent party. Both parties provided detailed descriptions of qualifying credentials. The study protocol was reviewed and approved by the Rowan University School of Osteopathic Medicine IRB (PRO-2023–131). All portions of the study were optional. Electronic informed consent was presented as the first portion of the study and obtained from all participants prior to participation. All data were anonymized throughout data collection and processing to ensure confidentiality. No protected, identifiable health information was collected.

## Results

A total of 124 individuals completed the survey, of which, 81 also concomitantly participated in at least 1 aspect of the health screening (blood pressure or blood sugar). The demographic composition of the survey participants (Table 1) was predominantly female (73.3%), middle-aged (*M* = 54.6 years), English survey preferred (84.7%), education of highschool or less (54.6%), with lower socioeconomic status based on income and insurance, who perceived their overall, dental, and mental health to be “Good”.

**Fig. 1:**
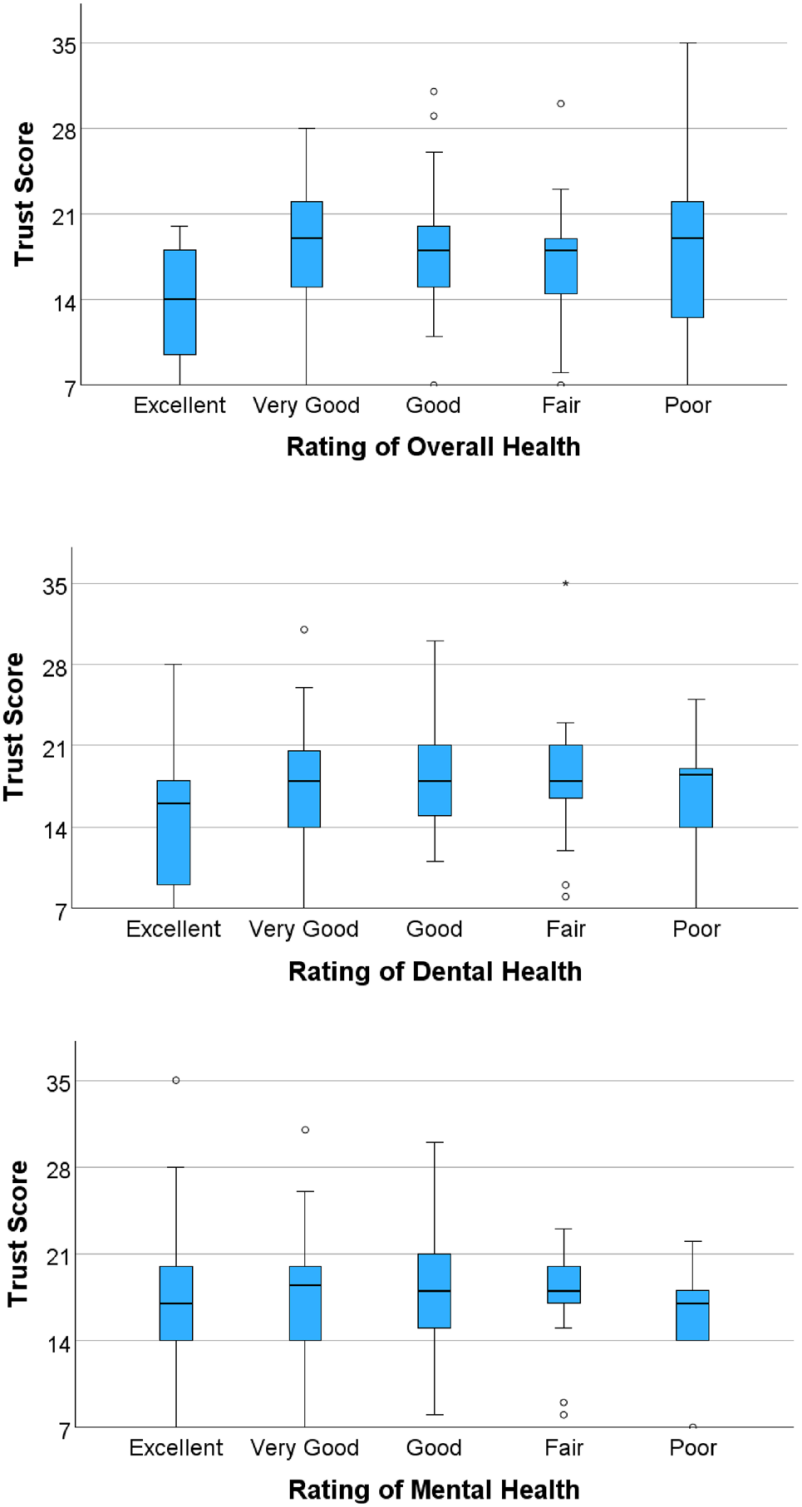
Participant self-perception of overall, dental, and mental health

Based on cumulative trust score, there was no statistically significant difference between participants based on a variety of demographic factors: region (defined by ZIP code), gender, age, income, completion in English or Spanish, if children lived at home, or the last visit to a doctor or dentist. From those who concomitantly completed the health screening, there was no correlation between trust and point of care blood glucose or blood pressure. In terms of self-perception of overall and dental health, participants who reported an “Excellent” rating were found to have significantly less trust than their counterparts. For overall health (Fig. 1a) in particular, participants reporting “Excellent” (*M* = 13.7, *SD* = 4.6) were statistically different from those who reported “Very Good” (*M* = 18.1, *SD* = 5.9, *p* =.014), “Good” (*M* = 17.9, *SD* = 4.3, *p* =.033), or “Fair” (*M* = 17.0, *SD* = 4.9, *p* =.043) perception. A similar trend was noted in those reporting “Excellent” (*M* = 14.6, *SD* = 5.5) dental health (Fig. 1b) to those who responded “Good” (*M* = 18.5, *SD* = 4.3, *p* =.034) and “Fair” (*M* = 18.5, *SD* = 5.0, *p* =.027). No additional statistically significant differences were identified between groups, including respondents to self-perception of mental health (Fig. 1c).

**Fig. 2:**
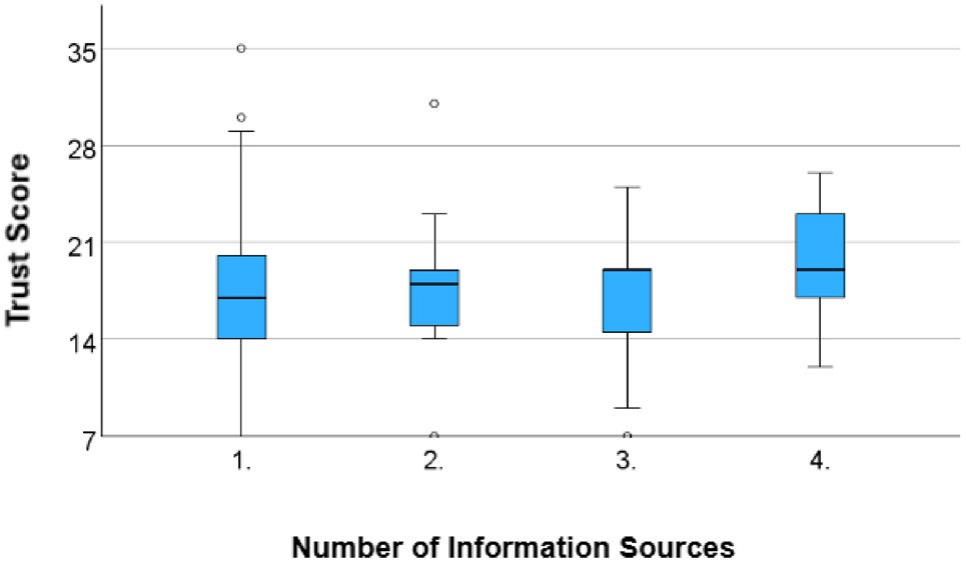
Total number of healthcare information sources reported by participants

Stratifying responses by reported sources of healthcare information, a majority of respondents cited their personal doctor or health care provider (*n* = 68), friends/relatives (*n* = 33), health insurance company (*n* = 22), or independent internet sources (*n* = 17) (Table 2). Only those who reported getting their healthcare information from “Books/magazines/newspaper” were found to have significantly more trust (*p* =.038) than those who did not, the sample size of this survey option was relatively small. Of note, while receiving healthcare information from one’s personal doctor was not statistically significant (*p* =.132), those who did receive information from their doctor (*M* = 17.8, *SD* = 5.6) had more trust than those who did not (*M* = 16.5, *SD* = 4.6). Participants'information seeking habits were further distilled into the total number of sources they reported using to learn about healthcare. Those reporting the use of 1 source had a mean of 16.82 (*SD* = 5.734) had a lower average trust than those who used 2, mean of 17.86 (*SD* = 4.475, *p* =.509), 3 (*M* = 17.33, *SD* = 4.865, *p* =.536), or ≥ 4 sources (*M* = 19.38, *SD* = 3.969, *p* =.086) of information (Fig. 2).

**Table 2:** Means of obtaining healthcare information (n = 118)

Source of healthcare info	Proportion		Trust score	*p*-value
	*n*	%	*M* (± *SD*)	
Email/online news subscriptions	5	4.2	21.2 (*SD* = 3.4)	.070
Federal and International health information sources (U.S. Department of Health, CDC, WHO, etc.)	9	7.6	19.6 (*SD* = 3.6)	.088
Books/magazines/newspaper	8	6.8	19.4 (*SD* = 2.3)	**.038**
Mobile apps (News app, Google, etc.)	9	7.6	19.2 (*SD* = 5)	.264
Television/Radio programs	12	10.2	19.1 (*SD* = 4.2)	.145
Independent internet sources (WebMD, Mayo Clinic, blogs, etc.)	17	14.4	18.5 (*SD* = 4.1)	.216
Social media	10	8.5	17.8 (*SD* = 3)	.576
Personal Doctor or health care provider	68	57.6	17.8 (*SD* = 5.6)	.132
Local, County, or NJ Department of Health (through their website, Community Health Worker, etc.)	13	11	17.6 (*SD* = 5.5	.784
Health insurance company	22	18.6	16.4 (*SD* = 4.1)	.356
Friends/relatives	33	28	16.7 (*SD* = 3.9)	.421
Work	7	5.9	16.7 (*SD* = 4.9)	.808

Bold indicates statistical significance (*p* < .05)

No significant difference in primary location healthcare services are received (Supplemental Table S2) was appreciated. The correlation between trust and reported preventative health measures was also explored (Table 3). Only annual flu vaccination was found to be statistically significant wherein those who had received their vaccination had higher average trust (*M* = 19.06, *SD* = 5.6) than those who had not (*M* = 16.05, *SD* = 4.7).

**Table 3:** Participation in annual preventative health measures

Place	Trust score (received)		Trust score (not received)		*p*-value
	*n*	*M* (± *SD*)	*n*	*M* (± *SD*)	
Flu Vaccination	48	19.1 (*SD* = 5.6)	76	16.1 (*SD* = 4.7)	**.002**
COVID Vaccination	59	17.7 (*SD* = 5.9)	65	16.8 (*SD* = 4.6)	.396
Hypertension Screening	76	17.3 (*SD* = 5.4)	48	17.0 (*SD* = 5.1)	.726
Cholesterol Screening	60	17.9 (*SD* = 4.9)	64	16.6 (*SD* = 5.5)	.215

Bold indicates statistical significance (*p* < .05)

## Discussion

This paper endeavors to examine relationships between medical mistrust and participant demographics, information seeking behaviors, preference of healthcare location to seek services, self-perception of health (overall, dental, and mental), and select preventative measures (annual flu/COVID vaccination and hypertension/cholesterol screening) in a medically underserved area. In existing literature, medical mistrust refers to the patient's relationship with clinicians and healthcare systems [24]. This study demonstrates the importance of locally evaluating systemic gaps in care to address upstream opportunities for impact. We identified that individuals who perceived their overall and dental health to be “Excellent” to have lower trust than their counterparts. The total number of different healthcare information sources reported to be consumed demonstrated that the greater total number of healthcare information sources used was positively correlated with trust. In terms of preventative measures, trust was found to be significantly higher in those who received an annual flu vaccination.

The relationship between one’s education and their own healthcare is multifaceted. Beyond educational attainment alone, patients being conscious of concerning symptoms has a direct impact on outcomes [25, 26]. In our study, educational attainment was not found to be significantly correlated with trust, however, other knowledge seeking behaviors were. Study results revealed that certain forms of media, “Books/magazines/newspaper”, may correlate with higher trust. Furthermore, a positive correlation was identified between the total number of healthcare information sources reported and medical trust. This trend was most apparent relative to participants who reported using only one source of media for healthcare information. In consideration of health literacy, it has been suggested that to improve trust in the healthcare system, there is a duality that must be acknowledged on the part of providers and patients [27]: (1) providers must effectively communicate concise information in a culturally sensitive manner [28] and (2) patient must take agency for their health and seek pertinent information for themselves and/or their community. The positive correlation in our study between diversity of healthcare sources and trust may exemplify the latter. As providers are offered the opportunity to serve as educators, positive interactions may strengthen relationships and build trust.

When it came to the primary location healthcare services are received (hospital ER, doctor’s office, urgent care, or clinic/healthcare center), there was no statistical significance based on trust alone which is in line with other studies in the literature. Given a population of economically disadvantaged individuals in the same community, Arnett et al. investigated differences in race and primary location for healthcare services. When controlling for mistrust and other demographic factors, they identified a significant difference in hospital outpatient department usage but not hospital ER or primary care office [12]. The sample size of our study prevented the use of logistic regression models to further analyze this relationship and control for similar variables.

Medical mistrust has been implicated as a negative effector of various preventative health measures [29, 30], with vaccine hesitancy being a particular area of focus [31–33]. Our results revealed that there was no significant relationship between medical mistrust and participants that did not receive annual COVID vaccine which is inconsistent with other research [33]. However, those who received annual flu vaccination had significantly greater trust which is supported by the literature [34]. Previous research investigated the potential correlation between trust, race, and discrimination on (1) feeling hesitant about COVID-19 vaccination and (2) prior COVID-19 vaccine acquisition [16]. The investigators showed that mistrust was associated with both vaccine hesitancy and acquisition, race with vaccine acquisition but not hesitancy, and perceived discrimination with neither [16]. Given the role of race in vaccine acquisition, future work may sample a larger, more diverse population in order for such factors to be detected in statistical analyses. It is also possible that given the publicity surrounding the COVID-19 pandemic, the time period of data collection, 2020 for Morgan et al., may make their results less generalizable to more contemporary data. Comparisons between Morgan et al. and the present work should be made with this consideration in mind.

Contextualizing the findings of medical mistrust and perception of health, it is plausible that those who have perceived their health to be “Excellent” may not engage in the same level of health knowledge seeking behaviors, or seek healthcare services in the same capacity as those who perceive their health as less than “Excellent”. Those who reported an “Excellent” perception of their overall and dental health were found to have significantly less trust than their counterparts. Similarly, another study showed that relative to non-hispanic whites, non-hispanic Blacks reported greater mistrust and lower proportions of self-perceived “Poor” or “Fair” health [12]. However, in their study, the analogous, non-hispanic white population was statistically older and ailed by more medical comorbidities. Therefore, the lower perception of health in the older population is confounded by these conditions and can not be attributed to race alone. In an incarcerated population, perception of health was found to be associated with the patient's primary source of healthcare information [35]. This suggests that the direct relationship between individuals'perception of health and trust may be more complex and confounded by race or source of healthcare information.

When investigating the impact that demographic factors have on trust and health outcomes, it is important to consider the generalizability of the findings, especially in the context of the study population. Idan et al. analyzed a sample of African American men and found insurance status to have a greater impact on trust relative to income and education [36]. Others have expanded on the role of discriminatory experiences and medical mistrust and found elements of class to be a largely associated effect modifier of discrimination [37]. Brown et al. also found higher income to be positively correlated with trust when controlling for experiences of discrimination. In the present study, differences in mistrust based on age, sex, geographic region, gender, income, race, or completion in Spanish or English were not identified. Given that our sample population is skewed to individuals of lower socioeconomic status, there is evidence to support that lower socioeconomic status may have mitigated some of the differences other studies have reported [37]. These comparisons may have also been limited by sample size.

While factors that contribute to medical mistrust, and the potential implications of this mistrust, have been broadly studied, there is conflicting evidence within the literature about which factors may be most significant. Building on previous work which has demonstrated the importance of physicians having culturally competent interactions, the current study results may suggest that trust is also influenced in a reciprocal manner by a patient's engagement in knowledge seeking behaviors. One means of bridging this gap is leveraging the support of community health workers who have filled the role of educators and liaisons for connecting the community to relevant health resources [38–40]. In a follow up to a community‐engaged research initiative (Forward Movement Project), researchers explored medical distrust in an underserved, rural community through participant-driven discussion with trained moderators [9]. Webb Hooper et al. (2022) reported positive data on the effectiveness of this intervention with participants subsequently being more likely to initiate dialogue with their providers. By improving communication and using concise language, all members of the care team can improve patient understanding and health outcomes [27]. While health information may be communicated in a variety of forms, ensuring that these resources are accessible [41] and culturally responsive [28] can further supplement this aim.

This research solicited the perceptions of healthcare trust and health behaviors of English and Spanish speaking individuals at the sub-county level. While county level data may provide broad insight into healthcare trends, understanding the barriers perceived by the community is a crucial component to characterize before interventions can take place. Private survey administration (via participants'phones or provided iPad) and keeping all responses fully anonymous were implemented to reduce potential social desirability bias. By allowing respondents more flexibility in skipping any question, sub-group analyses were limited by the overall sample size and completeness of responses. Additionally, comparisons could only be made between those who did or did not select a given option within a question as a result of question structure (i.e. allowing select all that apply rather than a ranked order or limited response type). This latter limitation applies to the comparison of medium for healthcare information consumption (Table 2), preventative screenings (Table 3), and primary location for healthcare services (Table S2).

The conclusions of this work may not be generalizable to other geographic regions where health systems, attitudes, or the population in general, do not align. Due to the nature of the subject matter, both vaccinations and medical mistrust, time period is especially important when comparing these results and can not be understated. More so, we hope that these results highlight the potential benefits of screening local communities at a more granular level to understand the specific needs of groups who may otherwise be blurred into an average of a county or state at large. Future work may investigate the variables presented in a targeted format to potentially increase sample size and survey completeness. Additionally, investigating a greater number of preventive health measures most relevant to the region may provide local providers insight into why these opportunities are being missed.

## Supplementary Information

Below is the link to the electronic supplementary material.

Supplementary file1 (DOCX 17 kb)
