# Supplementary material for: Influences and Implications of Medical Mistrust on Healthcare Behaviors in a Low Health Outcomes County in the State of New Jersey
Source: J Community Health. 2025 May 27;50(5):939–47. doi: 10.1007/s10900-025-01483-5 (PMC12474629; doi:10.1007/s10900-025-01483-5)
Supplement: Supplementary file 1 — Supplementary file1 (DOCX 17 kb) [file 10900_2025_1483_MOESM1_ESM.docx]

Article Title: Influences and Implications of Medical Mistrust on Healthcare Behaviors in a Low Health Outcomes County in the State of New Jersey

Journal Name: Journal of Community Health

Author Names: Dale Johnson*, MS, Adeena Javed, BS, Nathaniel Byrnes, BS, Anne Jones, DO, MPH, and Kristin Bertsch PhD

Affiliation: Department of Family Medicine, Rowan-Virtua School of Osteopathic Medicine, Stratford, NJ, USA

*Corresponding Author Email: johnso127@rowan.edu

***Table S1*** *Abridged Medical Mistrust Index Scale*

| # | Question |
| --- | --- |
| 1 | You'd better be cautious when dealing with health care organizations |
| 2 | Patients have sometimes been deceived or misled by health care organizations |
| 3 | When health care organizations make mistakes they usually cover it up |
| 4 | Health care organizations have sometimes done harmful experiments on patients without their knowledge |
| 5 | Health care organizations don't always keep your information totally private |
| 6 | Sometimes I wonder if health care organizations really know what they are doing |
| 7 | Mistakes are common in health care organizations |

***Table S2*** *Primary Location Healthcare Services are Sought (n=118)*

| Location | Proportion | | Trust Score | *p*-value |
| --- | --- | --- | --- | --- |
|  | *n* | % | *M* (± *SD*) |  |
| Hospital ER | 36 | 30.5 | 18.2 (*SD*= 5.7) | 0.240 |
| Doctor’s office | 71 | 60.2 | 17.6 (*SD*= 5.4) | 0.420 |
| Urgent Care | 46 | 39.0 | 17.3 (*SD*=5.3) | 0.909 |
| Clinic or healthcare center | 26 | 22.0 | 16.3 (*SD*=6.1) | 0.361 |
